# Tuberculosis treatment outcomes and associated factors among tuberculosis patients treated at healthcare facilities of Motta Town, Northwest Ethiopia: a five-year retrospective study

**DOI:** 10.1038/s41598-024-58080-0

**Published:** 2024-04-02

**Authors:** Liknaw Workie Limenh, Asmamaw Emagn Kasahun, Ashenafi Kibret Sendekie, Abdulwase Mohammed Seid, Melese Legesse Mitku, Eneyew Talie Fenta, Mihret Melese, Mulualem Workye, Wudneh Simegn, Wondim Ayenew

**Affiliations:** 1 Department of Pharmaceutics, School of Pharmacy, College of Medicine and Health Sciences, University of Gondar, Gondar, Ethiopia; 2 Department of Clinical Pharmacy, School of Pharmacy, College of Medicine and Health Sciences, University of Gondar, Gondar, Ethiopia; 3 Department of Pharmaceutical Chemistry, School of Pharmacy, College of Medicine and Health Sciences, University of Gondar, Gondar, Ethiopia; 4 Department of Public Health, College of Medicine and Health Science, Injibara University, Injibara, Ethiopia; 5 Department of Human Physiology, School of Medicine, College of Medicine and Health Sciences, University of Gondar, Gondar, Ethiopia; 6 Department of Pharmacy, College of Medicine and Health Sciences, Wollo University, Dessie, Ethiopia; 7 Department of Social and Administrative Pharmacy, School of Pharmacy, College of Medicine and Health Sciences, University of Gondar, Gondar, Ethiopia

**Keywords:** Tuberculosis, Treatment outcomes, Associated factors, Retrospective study, Motta town, Health facilities, Ethiopia, Microbiology, Medical research

## Abstract

Tuberculosis (TB) remains a significant public health concern, particularly in low-resource settings. The treatment outcome is a crucial indicator of the effectiveness of TB treatment programs. Assessing the current treatment outcome and its associated factors is essential for improving patient care and reducing the spread of TB. Therefore, this study aimed to assess TB treatment outcomes and their associated factors among TB patients who received treatment at public healthcare facilities in Motta Town, Northwest Ethiopia. A facility-based retrospective cross-sectional study design was employed in two TB treatment centers in Motta town from January 2017 to December 2021. The study participants were all patients diagnosed with TB who received treatment. A *p*-value of 0.05 with a 95% confidence interval (CI) was used to determine statistical significance. A total of 362 TB patients were included in the study. The overall treatment success rate was 88.4% (95% CI 85.1, 91.7). Male gender (AOR = 2.40, 95% CI 1.16, 4.98), normal nutritional status (AOR = 3.11, 95% CI 1.33, 7.25), HIV negative status (AOR = 3.35, 95% CI 1.31, 8.60), and non-presumptive drug resistance to TB (AOR = 3.72, 95% CI 1.74, 7.98) were significantly associated with successful TB treatment outcomes (*p* < 0.05). In the current study, nine out of ten study participants had successful TB treatment outcome rates. Male gender, normal nutritional status, non-presumed drug resistance to TB, and HIV-negative status were significantly associated with successful TB treatment outcomes. By taking risk factors associated with poor treatment outcomes like those found in this study into account, patient management and treatment can be optimized. Sufficient TB control measures for populations are imperative and could significantly reduce the nation's total TB burden.

## Introduction

Tuberculosis (TB) is a communicable disease that is a major cause of illness and death worldwide. Prior to the COVID-19 pandemic, TB surpassed human immunodeficiency virus/acquired immunodeficiency syndrome (HIV/AIDS) as the most common infectious agent-related cause of mortality^1^. It continues to be a significant worldwide health concern, although it is largely curable with treatment that is affordable and readily available^2–4^. Active TB can infect 5–15 people annually through close contact. The World Health Organization (WHO) Global TB Report 2022 shows a decrease in cases from 7.1 million in 2019 to 5.8 million in 2020, with a partial recovery in 2021 to 6.4 million. The COVID-19 pandemic disrupted TB healthcare services, leading to fewer diagnoses and treatment, increasing undiagnosed cases^1^. Geographically, TB burden varies, with the highest rates observed in regions such as Southeast Asia, Africa, and the Western Pacific. Eight countries accounted for a significant portion of global TB cases, with India being the most affected^5^.

Ethiopia is among the countries with a high prevalence of TB, TB/HIV co-infection, and multidrug-resistant TB (MDR-TB). Despite a drop in TB incidence and mortality rates in 2015, significant numbers of deaths, particularly among those co-infected with HIV, were recorded in subsequent years^3,6^. Improvements in living conditions, DOT applications, TB control initiatives, and global end-TB strategies have reduced TB rates. However, Ethiopia still faces a high disease burden, ranking 15th globally and 7th in Africa. In 2021, an estimated 143,000 new cases and 21,100 TB-related deaths occurred. DR TB was present in 2.7% of new cases and 14% of previously treated cases in Ethiopia in 2020^7^. Previously treated TB patients had an 8.1 times higher risk of developing MDR-TB compared to newly diagnosed TB patients^8^. Overall, 6.3% of MDR-TB isolates in Ethiopia's Amhara region were resistant to at least one second-line medication^9^.

In East Gojjam, Amhara region, 10.0% of previously treated cases and 15.58% of newly diagnosed cases showed drug resistance. Among retreatment cases, the prevalence of MDR-TB was 16.67%, significantly higher than the 1.29% in newly diagnosed cases^10^. In Motta General Hospital, the total prevalence of TB was 8.4%, while the prevalence of rifampicin resistance was 4.3% overall^11^.

The objectives of TB treatment include treating patients, halting the spread of the infection, and preventing the emergence of new DR strains. Nevertheless, these objectives are frequently not realized due to the seriousness of the illness, co-infection with HIV and/or other diseases, MDR, poverty, and the lack of care provided to patients^12^. Many people are not successfully treated for TB, even in areas where free medication is accessible. Incomplete treatment leads to an extended period of infection, DR, increased morbidity, and mortality. For 2018, the WHO sets a goal of 85% treatment success^13,14^. In 2020, the global success rate for TB treatment was 86%, almost unchanged from 2019. The TB incidence rate had to drop by 4–5% annually by 2020, 10% annually by 2025, and then an average of 17% annually from 2025 to 2035 in order to meet the milestones and targets for TB incidence reductions^1^.

Factors affecting TB outcomes are crucial indicators of TB control program success. Prompt initiation and adherence to national TB treatment standards are essential for effective therapy^15^. Monitoring the proportion of patients who receive successful treatment is key in evaluating the TB DOT program, particularly for those with TB and HIV co-infections due to potential treatment variables^6^. Understanding TB treatment outcomes is crucial, with categories like cure rate, completion, failure, death during treatment, and loss of follow-up assessing effectiveness. In Ethiopia, regional disparities show varying treatment success rates^16–18^. Challenges persist in Ethiopia, notably delays in TB diagnosis influenced by institutional and sociodemographic factors. Identifying and addressing gaps and weaknesses in the national TB control program is crucial for improving patient care and public health efforts^19^. Monitoring TB treatment effectiveness is vital for both clinical practice and surveillance purposes to enhance individual patient care and public health strategies^2^. Thus, this study aimed to assess TB treatment outcomes and their associated factors among TB patients who received treatment at public healthcare facilities in Motta Town, Northwest Ethiopia.

## Methods

### Study design

A facility-based cross-sectional retrospective study design was employed. The data was taken at the public health facilities using the 5 years patient record charts from January 1, 2017 to December 31, 2021.

### Study area and period

This study was conducted at public healthcare facilities in Motta town. Motta Town is one of the city administrations in the Amhara region. It is located in Amhara National Regional State, East Gojjam Zone, at a distance of 371 km from Addis Ababa and 120 km from Bahir Dar. The estimated total population in this town in 2007 was 26,177, of which 12,846 were females and 13,331 were males, according to data from the Central Statistical Agency. The town comprises one hospital and one health center, where TB patients receive diagnosis, treatment initiation, adherence support, and follow-up monitoring. The study was conducted from January 5 to 25, 2023. In these study facilities, the TB center is designed separately from other treatments. Both centers, especially the hospital, receive referrals from nearby rural health centers.

Ethiopia follows the WHO's TB treatment guidelines for diagnosis and management. Diagnostic techniques like Xpert MTB/RIF assays, microscopy, and culture confirm TB. Additional tests may be needed if bacteriologic methods are inconclusive. DR screening tests are recommended using Line Probe Assays or Xpert MTB/RIF. DOT is part of the National TB Program. Quick DST results guide treatment selection. Patients in contact with drug-resistant TB cases may start treatment based on source case DST results. EPTB is treated with the same six-month regimen as PTB, except for CNS and osteoarticular TB. A DR-TB diagnosis is crucial before starting second-line treatment. A shorter bedaquiline-based MDR-TB regimen is an option for certain patients^20^.

### Study population

The study included all TB diagnoses and treated patients with documented treatment outcomes and a thorough and legible record in the TB registration book. The study excluded patients who were referred out of study facilities.

### Sample size and sampling procedure

**Figure 1:**
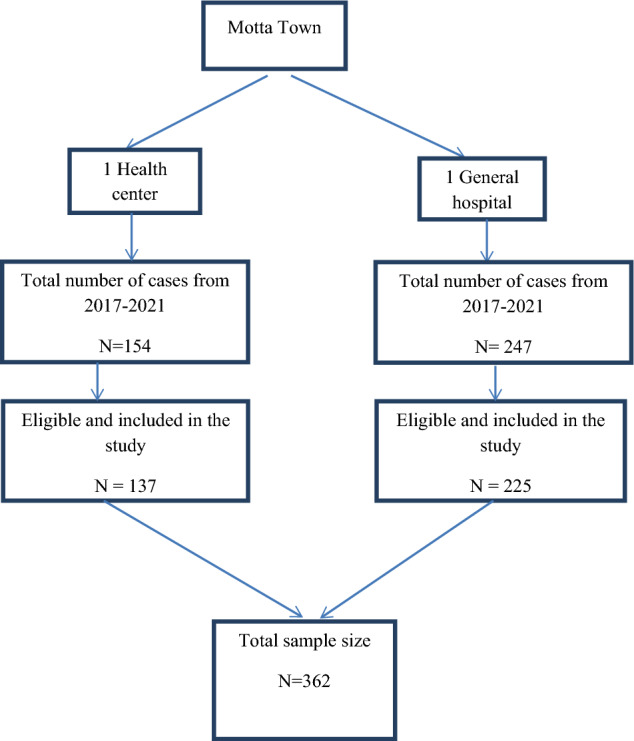
Sampling procedure among the study participants (n = 362).

Motta Town has one general hospital and one health center. These health facilities provided care for 401 TB patients between January 2017 and December 2021. Following that, the study included all of these patients whose information had been completed, and we also considered incomplete data as lost to follow-up (Fig. 1).

### Data collection procedure

To gather data on demographic characteristics and clinically relevant information, a structured data extraction form was developed. The TB patient registration record books were used to collect the data. Data were collected in the TB clinic by trained research assistants. The information gathered included the demographic (gender, age, weight, body mass index (BMI), target population, and TB risk group) and clinical (type of TB, TB category, expert MTB, Dx. expert result, nutritional assessment, HIV status, cotrimoxazole prevention therapy (CPT) started, antiretroviral therapy (ART) started, and presumptive DR) characteristics of TB patients as well as the results of their treatment.

### Variables

The dependent variables were TB treatment outcomes, and the independent variables were age, sex, weight, BMI, type of TB, category of TB, nutritional status, HIV status, presumptive DR, and patient categorization.

### Data processing and analysis

Each participant's data was entered into Epi Data version 4.2 and exported to SPSS version 25 for analysis. The sociodemographic and clinical features of the patients were summarized using descriptive statistics such as frequency, percentage, mean, and standard deviation. Bivariate and multivariate logistic regression models were used to analyze the associations between successful TB treatment outcomes and independent variables. A *p*-value less than 0.2 was used to select candidate variables for multivariate logistic regression. A *p*-value of 0.05 with a 95% confidence interval (CI) was used to determine statistical significance.

### Operational definitions

The National TB and Leprosy Control Program standard definitions and the 2021 modification of the WHO definitions and reporting framework for TB treatment outcomes^2,3,21–23^ used the following definitions:

**Cured**: A TB patient who started treatment with bacteriologically verified TB and finished the treatment as advised by national policy with evidence of a bacteriological response but no signs of failure.

**Treatment completed**: A TB patient who followed the national policy suggested a course of therapy but whose results did not fulfil the criteria for cure or treatment failure.

**Treatment failure**: A TB patient whose treatment plan required being stopped or permanently switched to another treatment plan.

**Died**: A TB patient who passed away for whatever reason, either before beginning treatment or while receiving it.

**Lost to follow-up**: A TB patient who has not started therapy or whose regimen has been stopped for eight or more weeks in a row after starting treatment at least four weeks previously.

**Not evaluated**: A TB patient for whom no treatment result has been established. This applies to situations where the reporting unit is unsure of the treatment outcome and situations where the case has been transferred to another treatment facility.

**New patients:** Patients with TB who have never undergone TB therapy or who have just started receiving anti-TB drugs. New patients may have positive or negative bacteriology and may have disease in any area of the body.

**Previously treated**: A TB patient may have the disease at any anatomical site, positive or negative bacteriology, and have taken anti-TB medications for at least one month in the past.

The following two treatment outcomes are classified by WHO standards^2^:

**Successful treatment outcome**: If TB patients finished therapy with symptom clearance or were cured (i.e., had a negative smear microscopy at the end of treatment and on at least one prior follow-up test).

**Unsuccessful treatment outcome**: If TB patients had treatment but had treatment failure (i.e., were still smear-positive after five months), lost to follow-up (i.e., patients who stopped taking their medication for two or more months consecutively after registering), or died.

### Ethical consideration

The study received ethical approval with a RefNo SOP 340/2022, from ethical review committee of the School of Pharmacy, College of Medicine and Health Sciences, University of Gondar. This committee also waived the need to obtain informed consent from the study participants because the data were obtained through a retrospective chart review. Before the data were collected, the hospital manager provided signed informed consent. All patient data will be kept confidential. The study methodology also complied with the Declaration of Helsinki. All shared-oriented information will be de-identified.

## Results

### Sociodemographic and related information

A total of 401 patients were enrolled in TB treatment in Motta Town. Of these 401 patients, 362 (90.3%) fulfilled our inclusion criteria and enrolled in this study. Males constituted the majority (58%) of the patients in the study. The mean age of the patients was 30, with a standard deviation (SD) of 14.3. The average patient weight was 47.14 kg, with an SD of 11.22. The average BMI for the respondents was 18.11 with a SD of 2.51. About 94.2% of the participants were anticipated to be general community patients (Table 1).

**Table 1:** Socio-Demographic and Clinical Profile of Tuberculosis Patients Registered at public healthcare facilities of Motta town, from 2017 to 2021 (n = 362).

Variables		Frequency	Percentage	Treatment outcome	
				Successful N (%)	Unsuccessful N (%)
`Gender	Male	210	58.0	193 (91.9)	17 (8.1)
	Female	152	42.0	127 (83.6)	25 (16.4)
Age	< 18	119	32.9	102 (85.7)	17 (14.3)
	18–44	90	24.9	83 (92.2)	7 (7.8)
	45–64	104	28.7	94 (90.4)	10 (9.6)
	> 64 years	49	13.5	41 (83.7)	8 (16.3)
Weight	< 30 kg	27	7.5	24 (88.9)	3 (11.1)
	30–50 kg	189	52.2	156 (82.5)	33 (17.5)
	> 50 kg	146	40.3	140 (95.9)	6 (4.1)
BMI	< 18.5	195	53.9	165 (84.6)	30 (15.4)
	18.5–22.9	150	41.4	144 (96.0)	6 (4.0)
	23–24.9	12	3.3	8 (66.7)	4 (33.3)
	> 25	5	1.4	3 (60.0)	2 (40.0)
Target population	General population	341	94.2	302 (88.6)	39 (11.4)
	Female commercial sex worker	3	0.8	3 (100.0)	0 (0.0)
	Prisoner	12	3.3	12 (100.0)	0 (0.0)
	Children of people living with HIV	6	1.7	3 (50.0)	3 (50.0)

*HIV* Human immunodeficiency virus, *BMI* Body mass index.

**Figure 2:**
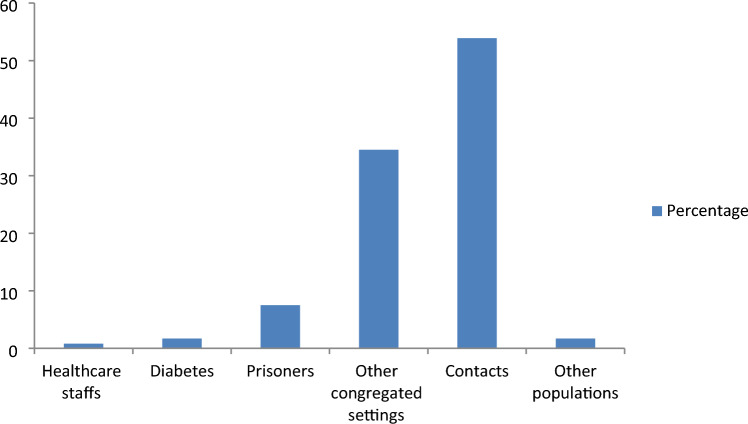
Tuberculosis risk group among the study participants (n = 362).

Most patients with TB (53.9%) were likely exposed to the disease through contact with an infected individual, while other congregated settings (living in a populated area) (34.5%) were the second-most risky category (Fig. 2).

### Clinical characteristics of tuberculosis patients

Only 35.4% of the patients had their TB initially identified with the Xpert MTB/RIF assay; the other patients were diagnosed with a different technique. About 87.6% of the patients were new cases. EPTB was present in 50.8% of patients. The nutritional assessment of the patients revealed that 61.9% of them had mild acute malnutrition (MAM). HIV testing was negative for about 88.3% of the patients. Among those who had an HIV test positive, 64.3% and 85.7% began CPT and ART, respectively. Inferred medication resistance was in about 4.4% of the patients (Table 2).

**Table 2:** Clinical characteristics of Tuberculosis patients at public healthcare facilities of Motta town, northwest Ethiopia from 2017 to 2021 (n = 362).

Variables		Frequency	Percentage
Expert MTB	TB patients diagnosed initially by X-pert	128	35.4
	TB patients diagnosed initially by other TB diagnosed method (AFB by direct microscopy, culture, and radiography)	234	64.6
Category	New case	317	87.6
	Transfer in	37	10.2
	Other previously treated patients	8	2.2
Type of TB	Pulmonary positive	109	30.1
	Extra-pulmonary	184	50.8
	Pulmonary negative	69	19.1
Nutritional status	MAM	224	61.9
	Normal	138	38.1
HIV status	HIV-negative	317	88.3
	HIV-positive	42	11.7
CPT Started	Yes	27	64.3
	No	15	35.7
ART started	Yes	36	85.7
	No	6	14.3
Presumptive DR	Yes	16	4.4
	No	346	95.6

*TB* Tuberculosis, *MTB Mycobacterium* tuberculosis, *AFB* Acid-fast bacillus, *HIV* Human immunodeficiency virus, *ART* antiretroviral therapy, *CPT* Co-trimoxazole preventive therapy, *DR* Drug resistance, *MAM* Moderate acute malnutrition.

### Tuberculosis treatment outcomes

**Figure 3:**
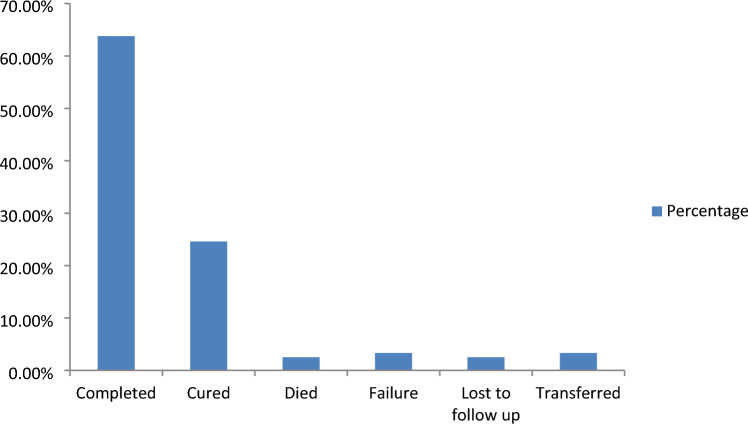
Tuberculosis treatment outcome among TB patients at public healthcare facilities of Motta town (n = 362).

The cure rate for TB patients who received TB treatment was 24.6%, while the completion rate was 63.8%. About 2.5% of the patients died. Three hundred and twenty (88.4%, 95% CI: 85.1, 91.7) TB patients had a successful TB treatment outcome in the current study (Fig. 3).

### Factors associated with tuberculosis treatment outcomes

In the bivariate analysis, gender, age, type of TB, category of cases, nutritional status, presumptive DR, and HIV status were the candidate variables for multiple logistic regression (*p* < 0.2). In the final model, being male, HIV negative, having non-presumptive DR TB, and having normal nutritional status were significantly associated with TB treatment outcomes (*p* < 0.05).

Male TB patients were 2.4 times (AOR = 2.40, 95% CI: 1.16, 4.98) more likely to achieve successful treatment outcomes than female TB patients. Regarding nutritional status, normal TB patients had 3.11 times (AOR = 3.11, 95% CI: 1.33, 7.25) higher chance of a successful treatment outcome than TB patients with MAM. In this study, HIV-negative TB patients were 3.35 times (AOR = 3.35, 95% CI: 1.31, 8.60) more likely to have successful treatment outcomes than HIV-positive TB patients. Non-presumptive DR TB patients were also 3.72 times (AOR = 3.72; 95% CI: 1.74, 7.98) more likely to have successful treatment outcomes than presumptive DR patients (Table 3).

**Table 3:** Bivariate and multivariate analysis of treatment outcome with socio-demographic and clinical characteristics of Tuberculosis patients attending healthcare facilities at Motta town, northwest Ethiopia, 2017 to 2221 (n = 362).

Variables		Treatment outcome		COR (95% CI)	AOR (95% CI)
		Successful N (%)	Unsuccessful N (%)		
Gender	Male	193 (91.9)	17 (8.1)	2.47 (1.16–4.31)	2.40 (1.16–4.98)*
	Female	127 (83.6)	25 (16.4)	1	1
Age	< 18	102 (85.7)	17 (14.3)	1.17 (0.47–2.92)	0.95 (0.34–2.62)
	18–44	83 (92.2)	7 (7.8)	2.31 (0.79- 6.82)	2.30 (0.72–7.37)
	45–64	94 (90.4)	10 (9.6)	1.83 (0.68–4.98)	2.06 (0.69–6.11)
	> 64	41 (83.7)	8 (16.3)	1	1
Type of TB	Pulmonary positive	100 (91.7)	9 (8.3)	2.34 (0.93–5.89)	2.09 (0.73–5.98)
	Extra-pulmonary	163 (88.6)	21 (11.4)	1.63 (0.76–3.53)	1.03 (0.40–2.65)
	Pulmonary negative	57 (82.6)	12 (17.4)	1	1
Category	New case	280 (88.3)	37 (11.7)	1.08 (0.13–9.04)	0.40 (0.04–4.14)
	Transfer in	33 (89.2)	4 (10.8)	1.18 (0.11–12.21)	0.39 (0.03–4.92)
	Other previously treated patients	7 (87.5)	1 (12.5)	1	1
Nutritional status	MAM	191 (85.3)	33 (14.7)	1	1
	Normal	129 (93.5)	9 (6.5)	2.48 (1.15–5.35)	3.11 (1.33–7.25)*
HIV status	HIV-negative	286 (90.2)	31 (9.8)	3.27 (1.50–7.15)	3.35 (1.31–8.60)*
	HIV-positive	31 (73.8)	11 (26.2)	1	1
Presumptive DR	Yes	13 (81.3)	3 (18.7)	1	1
	No	318 (91.9)	28 (8.1)	2.05 (1.56–5.78)	3.72 (1.74–7.98)*

*P < 0.05.

*TB* Tuberculosis, *HIV* Human immunodeficiency virus, *MAM* Moderate acute malnutrition, *DR* Drug resistance, *AOR* Average odd ratio, *COR* Crude odd ratio, *CI* Confidence interval.

## Discussion

The current study assessed TB treatment outcomes among treated patients to evaluate the effectiveness and efficiency of TB intervention programs. Most of the patients in our study were adults, and the majority of them were male, which was consistent with a study carried out at Woldia General Hospital in northeast Ethiopia^24^. This demographic pattern underscores potential gender disparities in TB detection and access to healthcare services. Moreover, the high proportion of adult patients suggests underlying socioeconomic factors such as poverty and limited healthcare access. These findings emphasize the importance of targeted TB intervention programs tailored to the specific needs of adult populations, particularly males, to improve TB awareness, early detection, and access to treatment.

The majority of the study patients were underweighted and generally had contact with people who were at risk for TB. The majority of our study participants were new patients, in line with a study done in Northern Ethiopia, where most patients (88.4%) were new cases^25^. These findings underscore the importance of targeting TB interventions towards individuals with known risk factors, such as undernutrition and close contact with TB-infected individuals. The predominance of new cases suggests ongoing transmission within the community and highlights the need for robust prevention strategies, early detection, and prompt treatment initiation to curb the spread of TB.

The majority (50.8%) of patients in our study were affected by EPTB, which was comparable with another study conducted in Northern Ethiopia, where 42.7% of study participants had EPTB^25^. On the other hand, a study carried out in Wolayta Sodo, Southern Ethiopia, discovered that most patients (65.9%) had PTB and only 34.1% had EPTB^26^. These variations in the distribution of TB types across different regions of Ethiopia highlight the heterogeneity of TB epidemiology within the country and may be attributed to various factors, including diagnostic challenges, immunocompromised populations, delayed presentation, and environmental factors. Studies have demonstrated that diagnosing EPTB often requires specialized techniques and clinical suspicion, leading to potential underreporting or misdiagnosis^27–29^. Moreover, individuals with compromised immune systems, such as those with HIV/AIDS, are more susceptible to EPTB^30–32^.

The higher number of EPTB cases than PTB cases could be caused by a number of factors. People who have both TB infection and HIV account for about 50% of EPTB cases^29^. The elderly, the homeless, those living in nursing homes, those with diabetes, those with impaired immune systems, malnourished people, people from low-income backgrounds, prisoners, alcoholics, children, people living in TB-endemic areas, and healthcare professionals are among the high-risk groups for EPTB^30,31^. Delays in diagnosis and treatment may arise from this, increasing the number of cases of EPT^32^. As malnutrition is common in the study area, this might be the reason for the high EPTB.

In our study, TB patients who received therapy had a higher (63.8%) completion rate than the cure rate. These results demonstrated that the majority of patients finished their treatment without having a sputum test. By starting the sputum test at the appropriate moment, stakeholders should take measurable action to increase the cure rate compared to the completion rate. It is concerning that some TB patients in our study as well as in many reports encountered unsuccessful TB treatment outcomes, although Ethiopia has a higher frequency of successful TB treatment results than previous estimates.

In the current study, more than four of five (88.4%, 95% CI: 85.1, 91.7) TB patients had successful treatment outcomes. This agrees with the studies conducted in Southeast Ethiopia (87.8%)^33^, in Northern Ethiopia (89.5%)^24^, Debre Tabor, Northwest Ethiopia (90.1%)^12^, and mapping TB treatment outcomes in Ethiopia (90.9%)^34^.

This result is higher than the studies conducted in Ghana (68.46%)^35^, North-Central Nigeria (67.4%)^36^, Somalia (85%)^15^, Wolayta Sodo, Southern Ethiopia (82.5%)^26^, Woldia, Northeast Ethiopia (80.7%)^24^, Adama City, Ethiopia (80.8%)^37^, Asella, Ethiopia (81.7%)^21^, Gambella, Southwest Ethiopia (70.76%)^38^, West Ethiopia (82.5%)^39^, Gedeo Zone, Southern Ethiopia (66.44%)^40^, Addis Abeba (82.7%)^13^, Ethiopian University Hospitals (60.1%)^41^, Dessie and Woldiya Town, Northeast Ethiopia (88.1%)^42^, and Debre Markos, Northwest Ethiopia (59.3%)^43^. Differences in sociodemographic characteristics, cultural practices, and socioeconomic status among these regions may contribute to variations in treatment outcomes.

However, the total treatment success rate of our findings was lower than that of a study conducted in Harar Town, Eastern Ethiopia (92.5%)^44^, Sekota Town, Northeast Ethiopia (93.8%)^45^, and West Gojjam Zone, Northwest Ethiopia (94.4%)^46^. These areas reported higher treatment success rates, likely attributable to superior healthcare infrastructure, better access to resources, and greater community involvement in TB control programs compared to Motta Town. These findings suggest that disparities in healthcare resources and community engagement may have contributed to the variation in treatment success rates across different regions within Ethiopia, highlighting the importance of tailored interventions and resource allocation based on local context.

Identifying the factors that contribute to the treatment outcomes of TB is essential to developing and recommending prompt solutions. The patient's gender was substantially related to the success of their TB treatments. Males were more than two times more likely to have successful TB treatment outcomes than females. This finding was supported by a study conducted in Woldia, Northeast Ethiopia, which found that male TB patients were 2.8 times more likely to have successful treatment outcomes than female patients [AOR: 2.8; 95% CI: 2.1, 4.8]^24^. However, these findings contrasted with a study conducted in Harar Town, Eastern Ethiopia, which found that female TB patients had a 1.89 times higher likelihood of getting a successful TB treatment outcome compared to male patients [AOR: 1.89, 95% CI: 1.14, 3.14]^44^. Sociocultural factors have been proposed as contributors to gender disparities in TB cases worldwide. Male patients’ treatment success rates may be influenced by factors such as stronger immune systems and fewer maternal-related challenges compared to females, potentially leading to a higher likelihood of successful outcomes^24^.

Additionally, there was a significant association between the patient's nutritional health and the success of the TB treatment in the current study. Patients with TB who have normal nutritional conditions are three times more likely to respond successfully to treatment. This is consistent with a study conducted in Addis Ababa, Ethiopia, which found that patients with normal nutritional status had a significantly higher likelihood of treatment success compared with those with poor nutritional status^47^. Biologically, malnutrition compromises the immune system's ability to mount an effective response against TB bacteria, leading to decreased treatment efficacy. Our study is also supported by a study conducted in Burkina Faso in which patients with malnutrition had a higher risk of treatment failure compared with those with normal nutritional status^48^. Another study on the impact of undernutrition on TB treatment outcomes in India found consistent evidence suggesting that malnourished patients with TB had a higher risk of unfavorable treatment outcomes, including treatment failure and mortality^49^.

The current study identified that TB patients who tested negative for HIV were more than three times more likely to have a successful treatment outcome. This finding is supported by a study conducted in Sekota Town, Northeast Ethiopia, which found that HIV-negative patients had a 2.64 success rate higher than HIV-positive individuals [AOR: 2.64, 95% CI: 1.20, 5.8]^45^. In addition, a study conducted in Harar Town, Eastern Ethiopia, revealed that HIV-negative patients had a success rate that was 6.50 times greater than HIV-positive patients [AOR: 6.502, 95% CI: 3.95, 10.71]^44^. On the other hand, this finding was less than that of research conducted in Adama Town, Central Oromia, Ethiopia, which found that HIV-negative patients were 20 times more likely than HIV-positives to experience a successful treatment outcome [AOR: 20.35, 95% CI: 7.73, 53.63]^50^.

HIV-positive patients experience poorer treatment outcomes compared to HIV-negative individuals due to HIV infection weakening the immune system, making individuals more susceptible to TB infection and progression to active disease^51^; delays in TB diagnosis and treatment initiation among HIV-positive individuals exacerbate disease progression and compromise treatment outcomes^27^; and complex drug interactions and adverse effects associated with TB-HIV co-treatment contribute to treatment interruptions and non-adherence, further compromising outcomes^52^.

The association between presumed DR and the success of TB treatment has been a topic of investigation in various studies. In this study, there was a significant association between presumed DR and the success of TB treatment. TB patients without presumptive DR had a success rate for their TB therapy that was more than three times higher than that of TB patients with presumptive DR. A study in Portugal explored the impact of drug resistance on TB treatment outcomes. The study found that patients without any drug resistance had significantly higher treatment success rates compared with those with drug-resistant TB^53^.

## Limitations

The use of retrospective secondary data, which is restricted to information recorded in the TB clinic's TB registers, was one of the study's key limitations. Besides the study design limitation, the database did not contain information on other potential factors, including compliance with treatment, duration of symptoms before treatment, distance travelled by patients to the health facilities, average CD4 count, type of DR, comorbidity, and health facilities' delays after TB patient diagnosis, all of which are known to be associated with TB treatment outcomes. Additionally, because these hospital and health center serve a limited geographic area, their patients may differ from those who live in other regions of the nation in terms of their demographics. Another limitation of the study design is that we are unable to draw causal conclusions about the effectiveness of the examined treatment, and the internal validity may be weak because of the absence of a comparator group.

## Conclusion and recommendations

More than four out of five patients with TB experienced a successful TB treatment outcome. Patients with TB who received therapy had a 24.6% cure rate and a 63.8% completion rate. Being male, HIV negative, free of presumptive DR, and in good nutritional health were all important factors in the successful outcome of TB treatment. Therefore, interventions targeting identified factors contributing to treatment outcomes, which include gender-sensitive approaches to TB care, enhanced nutritional support for malnourished patients, and integrated TB-HIV services to address the dual burden of disease, should be taken. A rapid molecular test (Xpert MTB/RIF) should be performed for fast diagnosis of TB and detection of resistance. Moreover, additional prospective studies are required to identify additional pertinent sociodemographic and behavioral characteristics that may influence how effectively TB patients respond to treatment.

## Data Availability

The research data generated and analyzed during the current study are available from the corresponding author upon reasonable request.

## Abbreviations

AIDS: Acquired immunodeficiency syndrome
AOR: Adjusted odds ratio
ART: Anti-retroviral therapy
BMI: Body mass index
CI: Confidence interval
COR: Crude odds ratio
CPT: Cotrimoxazole preventive therapy
DOT: Directly observed therapies
DST: Drug susceptibility testing
EPTB: Extrapulmonary tuberculosis
HIV: Human immunodeficiency virus
IP: Intensive phase
MAM: Moderate acute malnutrition
MDR: Multidrug resistance
M. TB: *Mycobacterium* Tuberculosis
PTB: Pulmonary tuberculosis
SD: Standard deviation
SPSS: Statistical Package for Social Science
TB: Tuberculosis
WHO: World Health Organization
XDR: Extensive drug resistance
